# Anal Cancer and Anal Intraepithelial Neoplasia Risk among Patients Treated for HPV-Related Gynecological Diseases—A Systematic Review

**DOI:** 10.3390/jcm12134216

**Published:** 2023-06-22

**Authors:** Michał Brzeziński, Maciej Stukan

**Affiliations:** 1 Department of Gynecological Oncology, Pomeranian Hospitals, 81-519 Gdynia, Poland; 2 Division of Oncological Propedeutics, Medical University of Gdańsk, 80-210 Gdańsk, Poland

**Keywords:** HPV, human papillomavirus, anal intraepithelial neoplasia, anal cancer, gynecology, cervix, vulva, vagina, intraepithelial neoplasia, cancer

## Abstract

Background: The most important causative agent of neoplasms in the anogenital area is the human papillomavirus (HPV). Due to the anatomical proximity of the genital and anus area and the ease with which HPV infection is transmitted, it seems that patients after the treatment of HPV-related gynecological diseases may have an increased risk of developing a second HPV-related neoplasm anal cancer. The aim of this study was to determine the risk of anal intraepithelial neoplasia (AIN) and anal cancer (AC) among patients after the treatment of HPV-related gynecological diseases. Methods: We conducted a comprehensive review of the available literature from multiple databases. The study was performed following *Cochrane Reviewers’ Handbook* and the Preferred Reporting Items for Systematic Reviews and Meta-Analyses 2009 guidelines. Moreover, we assessed the quality of each study using QUADAS-2. Results: Twenty-five studies were included in the final analysis. Patients after the treatment of HPV-related gynecological diseases have a significantly higher risk of AC (mean standardized incidence ratio (SIR) = 5.387, mean incidence risk (IR) = 0.096%, mean IR per 100,000 person–years = 10.37) and AIN (mean IR = 23.683%) compared to the population risk. Conclusions: patients with HPV-related gynecological diseases should constitute a group for which an appropriate primary and secondary screening for AC should be introduced.

## 1. Introduction

In 2020, there were 50,865 cases of anal cancer diagnosed worldwide. The cumulative incidence risk of this neoplasm is 0.07 for women and 0.06 for men [1]. HIV-positive homosexual men are the group with the highest risk of developing anal cancer (the incidence rate is 77–137 per 100,000) [2,3]. However, with regard to women, HIV infection is present in a relatively small percentage of AC patients. It is a chronic HPV infection that is the reason for developing nearly 90% of these neoplasms [4,5].

HPV-related gynecological diseases (HPV-RGDs) are those in which HPV plays a significant role in the pathophysiology. This group includes cervical cancer (CC), which is nearly entirely dependent on HPV infection, vaginal cancer (VaC) and vulvar cancer (VC), which are related to HPV infection in about 78% and 24.9% of cases, respectively [6], along with the corresponding precancerous lesions.

It has been investigated that HPV has the greatest affinity for zones where the epithelium of one type merges into another of a different histological feature, i.e., as in the cervix, where the squamous epithelium joins the one-layer glandular epithelium in the transformation zone [7]. HPV integrates its genome into the DNA of epithelial cells and expresses the E6 and E7 genes. Afterward, E6 and E7 proteins are produced, which initiate inter alia the degradation of p53 and Rb proteins. Finally, the processes characteristic of carcinogenesis are stimulated [8].

Another histologically similar region in the human body is the anal canal, where there is a transformation zone between the glandular and squamous epithelium at the level of the dentate line. Due to its anatomical proximity, this place seems to be particularly vulnerable to the transmission of HPV from infected gynecological organs and, as a result, susceptible to anal intraepithelial neoplasia (AIN) or anal cancer (AC) development. This has been supported by the data of Hernandez et al., which showed that in the case of Hawaiian patients with cervical HPV infection, 13% had concurrent anal HPV infection [9]. Moreover, Jacot-Guillarmod et al. showed that when cervical HPV infection was confirmed, simultaneously, anal HPV infection was presented in 59.3% of cases [10]. 

In the available research, HPV type 16 was responsible for infections in the perianal area and the anal canal in a subgroup of HIV-negative women in the overwhelming number of cases (about 85%). The second-most common was HPV type 18 followed by HPV types 31/33/45/52/58 [11]. The frequency and quality of HPV infections vary depending on whether there is a co-infection with human immunodeficiency virus (HIV). In the group of HIV-positive patients, the risk of anal cancer is significantly increased. Moreover, more diverse types of HPV can be found in rectal swabs [11]. However, one recent study showed that HIV-negative patients older than 45 with HPV type 16 detected in the cervix had a risk of developing anal cancer comparable to HIV-positive patients [12]. These data emphasize how substantially increased the risk of developing AIN/AC in patients with HPV infection in the anal canal is, especially with highly oncogenic HPV types. 

Considering all of the above facts, we formed a research hypothesis that patients diagnosed with HPV disease related to gynecological organs have an increased risk of developing AIN or AC due to the facility of the transmission of HPV infection into the anal canal.

The main aim of this study was to determine the risk of AIN/AC in HPV-RGD survivors. The secondary aim was to evaluate which HPV-RGD increases the risk of AIN/AC the most.

## 2. Materials and Methods

### 2.1. Search Strategy and Information Sources

In order to determine the risk of AIN/AC in the group of HPV-RGD survivors, a systematic review of the literature was performed in accordance with the criteria outlined in the Preferred Reporting Items for Systematic Reviews and Meta-Analyses (PRISMA) 2020 updated guidelines [13]. A review was carried out using the following databases: PubMed and EBSCO Discovery Service interface in order to search databases such as Medline, Cochrane Library, Web of Science, Academic Search Complete, ScienceDirect, Scopus, Nature Publishing Group, Oxford Journals, Wiley Online Library, and Clinical Key from 1992 to 1 November 2022. The search strategy, which included specific words and phrases (keywords), is enclosed in Supplementary Material File S1. 

### 2.2. Study Selection

The search strategy consisted of three stages, each conducted by both authors separately. In the first stage, we screened the titles using keywords enclosed in Supplementary Material File S1. In the second stage, we analyzed abstracts and, finally, in the third, the full text of previously selected articles regarding the inclusion and exclusion criteria. In case of disagreement about the inclusion of studies, a discussion was conducted between the authors.

### 2.3. Inclusion and Exclusion Criteria

Inclusion criteria: peer-reviewed prospective and retrospective cohort studies, randomized and non-randomized controlled trials; studies about the risk of AIN or AC presented as an indicator of standardized incidence ratio (SIR), incidence risk (IR), or IR per 100,000 person–years (PYs); studies where the analyzed group of participants were survivors of HPV-RGD; studies where the full text of the manuscript was available in English. 

Exclusion criteria: non-peer-reviewed publications, letters to the editors, systematic reviews, meta-analyses, case reports or case series; non-human subject studies; studies lacking adequate statistics (e.g., did not show separate results for AIN or AC or did not show separate results for specific HPV-RGD); studies where there were doubts whether the diagnosis of AIN/AC was confirmed histopathologically.

### 2.4. Data Extraction and Synthesis

Data were extracted by both authors independently. Extracted data included SIR (according to the following formula: SIR = observed cases/expected cases; see Supplementary Material File S3 for details providing definition and interpretation of SIR) and/or IR; in cases where the authors of the reviewed publication did not provide data, the IR was calculated as [number of new cases of disease during specified period]/[size of population at start of period] (see Supplementary Material File S4 for details providing definition and interpretation of IR) and/or IR per 100,000 person–years (PYs). In studies where authors did not provide information about IR per 100,000 PY, but provided data on PY, it was calculated as [number of new cases of disease] × 100,000/PY (see Supplementary Material File S5 for details providing definition and interpretation of IR per 100,000 PY). The obtained data were synthesized, and the mean was calculated for the risk of AIN and AC and for each HPV-RGD separately, with confidence intervals for a 95% level of uncertainty. The numbers of patients from all reviewed articles diagnosed with a given HPV-RGD were summed.

If the “carcinoma in situ” (CIS) term was separately used in publications, this was considered as cervical intraepithelial neoplasia (CIN) 3; thus, CIN3 and CIS were analyzed as one entity. Similarly, the term “low-grade squamous intraepithelial lesion” (LSIL) was considered equal to CIN 1, and “high-grade squamous intraepithelial lesion” (HSIL) was considered equal to CIN 2 or 3. This is in accordance with the current terminology [14].

When two or more publications used data from the same cancer registry and the period of research was similar, for final calculation, we selected data from only one of them (the most numerous), so as not to duplicate the number of patients with a given primary cancer/precancer.

### 2.5. Risk of Bias

To determine the bias risk, each article was assigned to a group of low, high, or unclear bias risk in accordance with the QUADAS-2 recommendations [15]. When determining the bias risk, the following factors were evaluated: patient selection, index test, reference standard, and flow and timing. Moreover, the same factors were evaluated except for flow and timing in case of determining the applicability of selected pieces of research. The exact process of determining bias risk and the applicability for each article is available in Supplementary Material File S2. Table 1 shows the general bias risk determined for each article included in the review.

## 3. Results

### 3.1. Search Results

**Figure 1:**
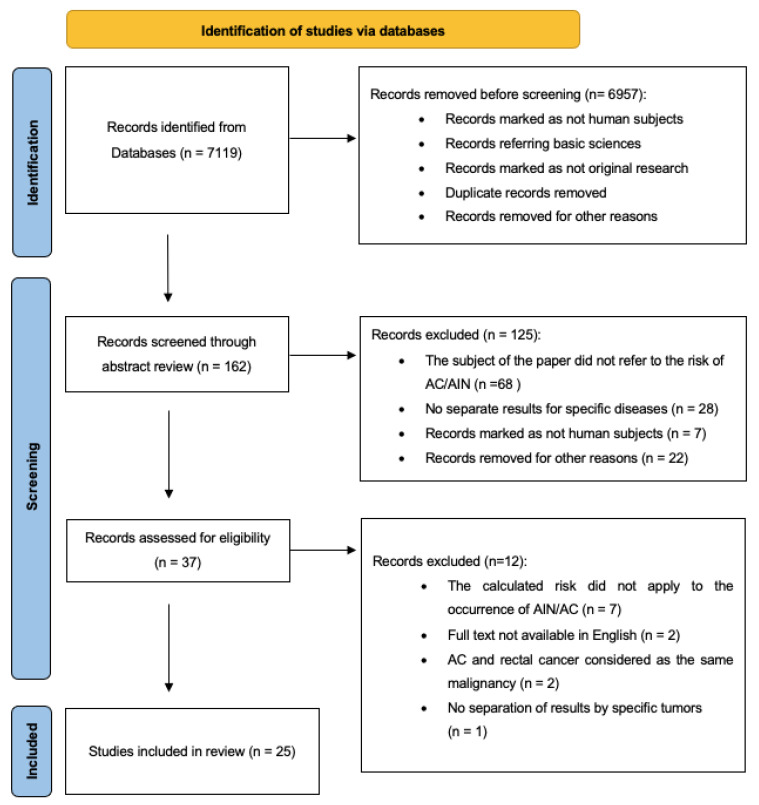
PRISMA flowchart summarizing the process for the identification of eligible articles. AC—anal cancer; AIN—anal intraepithelial neoplasia.

The search resulted in 7119 records. After the first stage of screening, six thousand nine hundred and fifty-seven records were excluded because of being marked as not human subjects, referring to basic sciences, different types of research than the original research, referring to other malignancies than AIN/AC, or being duplicated. Additionally, one hundred and twenty-five records were excluded after the second stage of screening because the subject of research did not concern the risk of AIN/AC, the results for different diseases were not divided, or the reasons mentioned in the previous stage of screening. Finally, after reviewing the full text of the selected articles, we included 25 articles in our research [17,18,19,20,21,22,23,24,25,26,27,28,29,30,31,32,33,34,35,36,37,38,39,40,41]. The reference list of the identified articles was reviewed, but no relevant studies were additionally added. There was no disagreement about the inclusion of the selected studies between the authors. The process of the study selection is shown in a PRISMA flowchart in Figure 1.

### 3.2. Study Characteristics

The study characteristics of the selected articles are presented in Table 1. There were 3 prospective cross-sectional studies and 22 retrospective population-based cohort studies. Only four articles determined the risk of AIN in patients with HPV-RGD, the rest of the articles (n = 22) determined the risk of AC. The mean age of patients at the diagnosis of CC was 57.7; VC, 65.5; and VaC, 81.

The total number of people diagnosed with CC was 177,984; VC, 9572; and VaC, 2733. In the case of precancerous lesions, there were 110,243 patients after the treatment of CIN 1; 52,151 after the treatment of CIN 2; 447,739 after the treatment of CIN 3; 234 after the treatment of VIN 1; 16 after the treatment of VIN 2; 108 after the treatment of VIN 3; 104 after the treatment of VaIN 1; 13 after the treatment of VaIN 2; and 30 after the treatment of VaIN 3. In several articles, the authors did not divide precancerous lesions into particular subcategories, and therefore, the total number of cases of CIN, VIN, and VaIN was counted. The total number of patients with a medical history of CIN was 682,991 (summed number of patients with CIN 1, 2, and 3, and the number of patients who were referred to in the selected publications as CIN, without specifying the degree of intraepithelial neoplasia). The total number of patients with a medical history of VIN was 358 and with VaIN was 147. 

### 3.3. Data Extraction and Synthesis Results

Several of the US studies analyzed data from a similar period from the National Cancer Institute Surveillance, Epidemiology, and End Results (SEER) Program. Moreover, two publications acquired data from the Finish Cancer Registry and three from the Swedish Cancer Register. In these cases, for the final calculation, we used only data from one publication (the one with the most cases). Additionally, data from Chaturvedi et al.’s publication were not included in the final calculation because they are based on 13 popu-lation-based cancer registries in Denmark, Finland, Norway, Sweden, and the United States—in this case, the risk of the duplication of patients was too high.

**Standardized incidence ratio (SIR)**. The mean SIR for AC in CC survivors was 3.814 (95% Cl: 1.21–6.41). The mean SIR for AC in VC survivors was 14.55 (95% Cl: 0.15–24.4). The SIR for AC in VaC survivors was 1.8 (95% CI: 0.2–5.3) (data from the research of Aceve-do-Fontánez et al. were not included, as the authors only reported a summary SIR for VC and VaC). The mean SIR for AC in CIN 3 survivors was 5.701 (95% Cl: 2.23–19.2). The mean SIR for AC in CIN 2 survivors was 1.895 (95% Cl: 0.09–4.1). There was no information about SIR for CIN 1, VIN, or VaIN survivors in any of the articles we reviewed. The mean SIR for AC in CIN survivors, without dividing into specific degree of intraepithelial neoplasia, was 4.563 (95% Cl: 0.12–19.2). The mean SIR for all HPV-RGD survivors was 5.387 (95% Cl: 2.99–7.78). Only one study provided data about SIR for AIN and it was 6.68 (95% Cl: 3.64–12.25) in CIN 3 survivors. 

**Incidence risk (IR).** In four studies, the authors calculated the IR [20,25,29,39]. For the remaining papers, we calculated the IR based on the data provided in the articles. Table 2 shows the results. The mean IR of AC in CC survivors was 0.086% (95% Cl: 0.07–0.102%); in VC survivors, it was 0.265% (95% Cl: 0.17–0.36%); in VaC survivors, it was 0.096% (95% Cl: 0.009–0.183%); in CIN 3 survivors, it was 0.084% (95% Cl: 0.076–0.092%); in CIN 2 survivors, it was 0.061% (95% Cl: 0.039–0.083%); in CIN 1 survivors was 0.013% (95% Cl: 0.006–0.02%); in CIN survivors (without dividing into subcategories), it was 0.066% (95% Cl: 0.06%–0.072%); in VaIN 3 survivors, it was 0.342% (95% Cl: 0.037–0.647%); and in VIN 3 survivors, it was 0.810% (95% Cl: 0.59–1.03%). We did not find any study that analyzed IR of AC in VaIN 1 or 2 and VIN 1 or 2 survivors. The mean IR of AC for all HPV-RGD survivors was 0.096% (95% Cl: 0.09–0.102%). The IR of AIN in CIN survivors was 16.45% (95% Cl: 13.25–19.65%); in VIN survivors, it was 36.4% (95% Cl: 28–44.8%); and in VaIN survivors, it was 18.2% (95% Cl: 7.8–28.6%). The mean IR of AIN for all HPV-RGD survivors was 23.683% (95% Cl: 20.55–26.82%).

**Incidence risk per 100,000 person–years (IR per 100,000 PY).** The mean IR per 100,000 PY of AC in CC survivors was 9.73 (95% Cl: 8.03–11.43); in VC survivors, it was 37.98 (95% Cl: 22.64–53.32); in VaC survivors, it was 11.78 (95% Cl: 0–29.15); in CIN 3 survivors, it was 5.78 (95% Cl: 5.18–6.38); and in CIN (1–3) survivors, it was 5.37 (95% Cl: 4.85–5.89). The mean IR per 100,000 PY of AC for all HPV-RGD survivors was 10.37 (95% Cl: 9.66–11.08). Only one study had sufficient data to calculate IR per 100,000 PY of AIN, a paper by Ebisch et al., where the risk of AIN 3 in CIN 3 survivors was 6.34 (95% Cl: 5.10–7.90). Table 2 shows the results.

The summarized results of SIR, IR, and IR per 100,000 PY of AC and AIN after primary diagnosis of specific HPV-RGD are presented in Table 3.

## 4. Discussion

In our study, we confirmed that survivors of HPV-RGD have a higher risk of both AIN and AC in comparison to the general population. Survivors of VC have the highest risk of developing AC, followed by CC survivors and VaC survivors. The risk of developing AC in patients with a history of CIN increased with the severity of CIN—the highest was for CIN 3 and the lowest was for CIN 1.

We hypothesize that the risk for VC survivors might be higher than for CC survivors because current CC treatments, mostly including surgical approaches, usually result in total HPV elimination. Furthermore, HPV subtype differences may also be significant. A study by Saraiya et al. showed that the spectrum of the most common HPV subtypes and their proportions detected in the tissues of these cancers differed [41].

Data on the risk of developing AIN after HPV-RGD are much scarcer compared to AC data. Only four studies analyzed the risk of developing AIN after HPV-RGD; in addition, all of them concentrated on patients with precancerous lesions: CIN, VIN, and VaIN [18,20,25,38]. We have not found any study that analyzes the risk of AIN after HPV-related gynecological neoplasms, i.e., CC, VC, and VaC. Research by Ebisch et al. was the only one that determined both the SIR and IR per 100,000 PY for AIN in patients with a prior diagnosis of CIN 3 [18].

Although we have far less data on the risk of AIN in people with HPV-RGD than on AC, it is evident that the risk of AIN in this subset of patients is substantially higher than AC because HSIL (AIN) is a direct precursor of AC. The large difference between the risk of AIN and AC may be due to the fact that only about one in ten people with AIN develop AC. In a study by Watson et al., on a high-risk group of 72 people with AIN, it was found that 11% of them went to AC in a median time of 42 months. What is more, approximately one-third of this population experienced a decrease in the stage or regression of the disease [42].

The greatest risk of AIN is in the case of a previous diagnosis of VIN, followed by VaIN and CIN. This is most likely owing to the existence of a well-functioning screening for CC, which detects CIN lesions, usually treated using excision of lesions, resulting in HPV elimination [43]. Because VIN and VaIN are less common, their diagnostic methods are not so well developed, allowing for longer-term HPV infection and its easier spread to the anogenital zone. The risk calculated by us is slightly higher than in a study by Santos et al.; however, the authors provided a cumulative AIN risk for CIN, VIN, and VaIN without separating them into individual types of precancerous lesions [44]. Moreover, Clark et al. proved that the actual prevalence of AIN may be even higher if high-resolution anoscopy (HRA) was used to detect disease because it has the highest sensitivity in detecting precancerous anal lesions [45].

In our study review, we showed that patients with a history of HPV-related disease have a significantly increased risk of AIN and AC. These data lead to the concept that these patients, after being treated for the previous HPV-related disease, should be strictly controlled to prevent the development of another HPV-related disease. Unfortunately, currently, there are no recommendations or guidelines that clearly define how such a control should look, how long it should last, and who should be responsible for it. Recently, the International Anal Neoplasia Society (IANS) assembled a Task Force in order to systematize and establish recommendations for AC screening [46].

We think that gynecologists should be responsible for preventing the development of HPV-related disease in the whole anogenital region. A gynecologist is a specialist to whom women regularly schedule for tests of the secondary prevention of CC—cytology and/or a test for high-risk human papillomavirus (hrHPV). The gynecologist takes a swab from the cervix. It is a quick and painless examination, which provides many benefits to patients. More importantly, in the context of this divagation, it is also a perfect moment when a gynecologist can perform a similar procedure and take an additional swab from the anal canal in a group of patients with a particularly high risk of development of AC. Thus, the gynecologist will become the person responsible for detecting such changes and then referring the patient to an anorectal disease specialist in order to implement tertiary prevention.

Another important issue that should be raised by gynecologists, in terms of the prevention of other HPV-related diseases, is prophylactic vaccination against HPV. This is applicable even in people of older ages and with HPV-related diseases. It has been shown to be effective in older ages [47,48] and in preventing recurrent CIN [49,50,51,52]. We believe that, in this case, it is also the gynecologist who should be responsible for disseminating knowledge and encouraging patients to be vaccinated against HPV. Currently, 9-valent, 4-valent, and 2-valent HPV vaccines have been licensed and are available. They are highly effective in preventing HPV infection and the following precancers and cancers of the cervix, vagina, vulva, anus, and, probably, also the oropharyngeal region attributable to types of HPV targeted by the vaccines [53,54,55,56,57,58,59,60,61,62,63].

**Figure 2:**
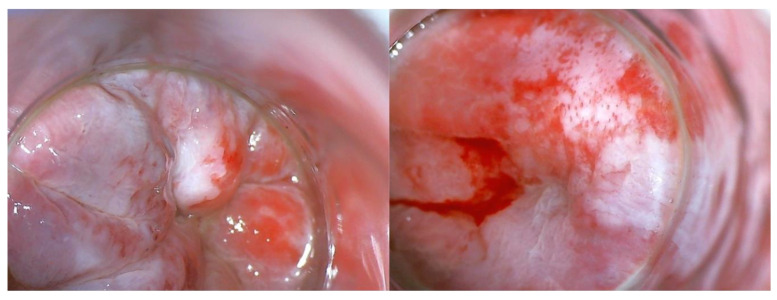
Anal mucosa seen with high-resolution anoscopy (HRA). Whitening of the anal mucosa after application of acetic acid indicates the probable location of anal intraepithelial neoplasia.

Currently, the gold standard in AC screening is HRA [64] (Figure 2). This allows the visualization of the rectal mucosa using an anoscope and the identification of macroscopic lesions that can be biopsied or locally excised and sent for histopathological examination. Due to the fact that in the anus, similarly as in the cervix, there is a transformation zone where glandular epithelium connects with squamous epithelium, tests that are used routinely in the screening of CC, i.e., cytology or hrHPV, may also be appropriate here. There are studies that compare the effectiveness of both methods in the context of AIN/AC diagnostics, but at the moment, it has not been determined which of the tests alone or in combination with other methods and for what population (probably for a pre-specified population at higher risk) would be ideal as an element of screening [11,45,65].

Due to the rarity of AC and economic issues, the introduction of routine AC screening, in the case of every patient who has ever been diagnosed with HPV-related cancer or a corresponding precancerous lesion, is impossible even for the richest countries. Therefore, it is important to select the subgroup of patients with the highest risk of AIN/AC and establish cost-effective algorithms concerning AC screening. To do this, the relevant risk factors must be identified.

The major issue is the identification of people with disorders in the functioning of the immune system. Patients suffering from HIV, in immunosuppression, or with congenital immunodeficiency disorders constitute a group wherein HPV infection occurs more often and spreads more easily. In this particular group of patients, the time to the development of AIN/AC will be much shorter than in people with a properly functioning immune system [66,67]. In programs for cervical cancer screening, patients with immunosuppression constitute a special population with distinct management compared to the general population [68,69]. In addition, there are data showing that in the case of HIV-positive people, the proportions of occurrence of different types of HPV in the anal canal are different compared to HIV-negative people. The frequency of infection with type 16 is decreased in favor of type 18. Moreover, the frequency of infection with types 31, 33, 45, 52, and 58 is also significantly increased. This is probably due to the greater ability of type 16 to evade host immune control compared to other types. In the case of deficiency of the immune system, infection with other types of HPV is able to survive longer and, thus, cause precancerous lesions followed by AC [11]. The population of people living with HIV might also benefit from HPV vaccination because its effectiveness was shown, especially for those with optimal CD4 cell count [70,71,72].

Surprisingly, only four of the studies that we have reviewed reported the HIV status of the research participants [20,25,27,38]. The authors of this research are convinced that, due to such a distinct pathophysiology of HPV infection in HIV-positive and HIV-negative patients, these two groups should always be analyzed separately in the context of subjects as discussed in this publication.

Another factor that should be mentioned is the time between the clinical disease and the onset of HPV infection. It has been proven that the risk of CC increases with increasing time since infection in the cervix. The median progression time from CIN 1 to CIN 2/3 is 2–3 years [73], and the subsequent median time to develop CC is 10–12 year [74]. Similar dependence can be found in the case of AC. Patients with long untreated active HPV infection have a higher risk of developing AIN/AC; however, the specific average amount of time required to develop AC has not yet been specified. It is also important to emphasize the fact that the treatment of an HPV-related lesion in a gynecological organ, e.g., CIN, does not mean the complete treatment of HPV infection. In the case of a long-term infection of the cervix, there is a high risk of transfer of HPV to nearby anatomical regions, e.g., to the anal canal. Such a patient, even after the complete treatment of gynecological intraepithelial neoplasia, may develop HPV-related diseases in the anal canal in the future. This is another argument for the need for AC screening in the subpopulation of people with the highest risk of developing HPV-related diseases.

Moreover, the specific type of HPV is another important issue in the case of analyzing the risk of the carcinogenesis process. The time needed for the development of CC in the case of high-oncogenic HPV types (mostly 16 and 18, but also 31, 33, 35, 39, 45, 51, 52, 56, 58, 59, 66, and 68) is significantly shorter in comparison with low-oncogenic HPV types. In the case of perianal infection and the development of AC, the pathophysiology is probably similar. However, further research is required to isolate the HPV types with the highest risk of carcinogenesis in the anal canal.

The age of the patient is also a valid risk factor. The incidence of AC increases by 2.7% per year, with pronounced increases in age groups 50 years and older [75]. The average life expectancy in the world is constantly increasing; therefore, the percentage of patients who live with various chronic diseases is also increasing. In the past, such diseases did not develop enough to be a direct cause of death. With advances in medicine and better awareness of the public about their own health, such diseases have become a significant public problem. An example is AC, a disease that is more common in older people. As the age of the population increases, the mortality rate due to this cancer increases. HPV types can be detected in 80–90% of all AC cases, which makes this neoplasm, second after CC, the closest HPV-associated cancer [76]. Due to the fact that, as in the case of CC, an appropriate amount of time must pass for the process of carcinogenesis to occur, it is young and middle-aged patients who will obtain the greatest benefits from the introduction of secondary AC prevention programs because the potential number of years gained after treatment of detected AIN is the highest. Bearing in mind the fact that society is constantly aging, we should already be ahead of the health problems in society that the future will bring us.

**Figure 3:**
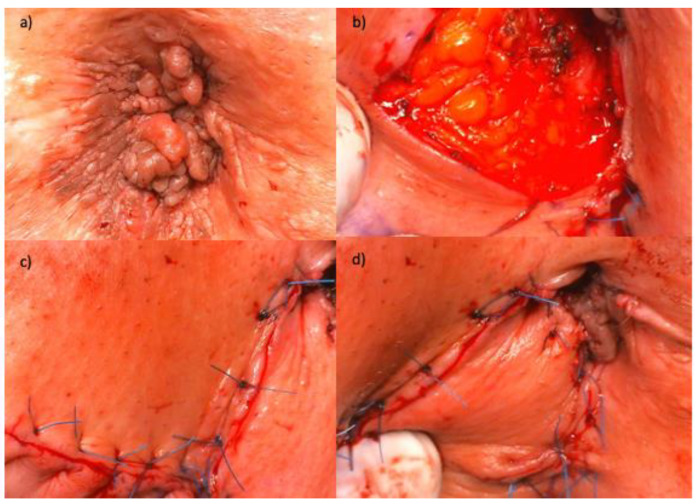
Surgical treatment of HSIL (AIN3) in perianal region. (**a**) Anal region with AIN before surgery. (**b**) Anal region after excision of AIN. (**c**,**d**) Anal region after reconstruction requiring skin grafts (Limberg flap). AIN—anal intraepithelial neoplasia.

Another major reason why additional screening in people with HPV-related diseases is important are the problems with the selection of an appropriate method of treatment in people with HPV-related extensive changes in the anogenital area. In such cases, treatment with topical cidofovir or imiquimod may be offered. Unfortunately, their effectiveness is quite low, and relatively, many patients do not tolerate treatment in maximum doses [77,78]. Surgical treatment is an alternative; however, it is used in limited lesions. In advanced lesions, surgery can be a mutilating procedure involving extensive operations sometimes requiring skin grafts from other areas (Figure 3).

The ANCHOR study has proven that the treatment of AIN reduces the risk of future AC in people living with HIV [79]. The next step should be to determine whether such a treatment is also beneficial in the HIV-negative population, i.e., in patients with HPV-RGD. However, the lack of clearly defined rules for the detection of AIN, especially in populations most at risk of developing AC, constitutes an obstruction. Therefore, the goal for the coming years should be to set clear, strictly defined rules of what AIN diagnostics should look like. 

The strength of our study is that we performed a systematic review of all relevant publications referring to the subject of research—not only the risk of AC but also what is unique and the risk of AIN in patients who were diagnosed and treated for gynecological HPV-related diseases. Additionally, a critical evaluation of the included articles was performed to provide a level of risk of bias. A summary of all available up-to-date data was also provided. 

We acknowledge the limitations of this study. The first is that the majority of evaluated publications were based on national registry databases. This kind of research is vulnerable to bias because the potential to under-report or misclassify diseases and procedures is possible. The second is that AC is a rare disease. Moreover, AIN might be significantly under-reported because there are no established screening programs, and HRA, as the best tool to diagnose AIN, is not easily available and is a difficult procedure. Low numbers of both AC and AIN might have a significant impact on the calculation of SIR. The smaller the number of cases of the target disease is, the less precise the SIR calculation is [80]. The third limitation is that the interpretation of data might be biased because of a relatively long time between the diagnosis of HPV-RGD and secondary AC, which is about 13.5 years from the diagnosis of cervical cancer, based on available data [29]. Thus, a diagnosis of AIN could be that the outcome should be considered as an end-point for future research evaluating the risk of anal HPV disease in a population of patients with HPV-RGD. Given the above-mentioned fact of the relatively long period of time between cervical cancer and the diagnosis of AC, the fourth obstacle should be recognized in terms of the interpretation of available data. Establishing the risk of secondary AC and its morbidity and mortality seems more important for a subgroup of patients treated for localized cervical cancer because over 90% of these patients survive longer than 5 years. Contrarily, only about 60% and 17% of patients with regional and distant disease, respectively, live longer than 5 years [81]. Again, the SIR number can be underestimated, though this issue might not be clinically useful, especially among patients with advanced, aggressive cervical carcinomas. 

## 5. Conclusions

The risk of developing secondary AC/AIN is significantly higher in groups of people with primary HPV-RGD than in the general population. Patients who have been diagnosed with VC have the highest risk of secondary AC/AIN. The risk of AIN is much higher in people with HPV-RGD than the risk of AC. Further studies are required to determine the exact risk of AIN in the HPV-RGD subpopulation, particularly in those with CC, VC, and VaIN. Targeted screening programs, including both surveillance (HRA, smears for hrHPV, and cytology) and HPV vaccinations, for AC/AIN, should be developed for patients with HPV-RGD. The true burden of AC/AIN can be determined in prospective studies addressing these specific programs. 

## Figures and Tables

**Table 1:** Summary of reviews about the risk of AIN/AC in groups of patients with HPV-RGD.

No.	Authors and Year of Publication	Geographic Location	Study Design	Sample Size	Period of Time Analyzed	The Type of Primary Cancer/Precancer Lesion	Median Age of Participants	Number of AIN/AC	Increased Risk	Risk of Bias
1.	Acevedo-Fontánez et al. (2018) [16]	Puerto Rico	Retrospective, population-based cohort study	9489	1987–2013	8039 CC 1378 VC 773 VaC	46, CC 70, VC 67, VaC	14 AC after CC 3 AC after VC 1 AC after VaC	AC after CC: SIR = 1.8 (95% Cl: 0.9–3.4) AC after VC and VaC: SIR = 2.9 (95% Cl: 0.8–7.5)	L
2.	Chaturvedi et al. (2007) [17]	Denmark, Finland, Norway, Sweden, and USA *	Retrospective, population-based cohort study	27,466	1973–2001	27,466 CC **	48.6	NI	AC after CC: SIR = 3.12 (95% Cl: 1.88–4.88)	L
3.	Ebisch et al. (2017) [18]	Netherlands	Retrospective, population-based cohort study	89,018	1990–2015	89,018 CIN 3	36	73 AC 80 AIN 3	AC after CIN 3: SIR = 3.85 (95% CI: 2.32–6.37) AIN 3 after CIN 3: SIR= 6.68 (95% CI: 3.64–12.25)	L
4.	Edgren et al. (2007) [19]	Sweden	Retrospective, population-based cohort study	125,292	1968–2004	125,292 CIN 3	35	131 AC	AC after CIN 3: SIR = 4.68 (95% Cl: 3.87–5.62)	L
5.	ElNaggar et al. (2013) [20]	Memphis, Tennessee (USA)	Prospective, cross-sectional study	272	2006–2010	CIN 1 29 CIN 2 16 CIN 3/CIS 41 VIN 1 46 VIN 2 16 VIN 3/CIS 69 VaIN 1 34 VaIN 2 13 VaIN 3/CIS 8 = 272	39	64 AIN (36 stage 1, 6 stage 2, 22 stage 3)	48 (36.4%) had VIN, 10 (18.2%) had VaIN, and 13 (14.4%) had CIN	H
6.	Evans et al. (2003) [21]	Southeast England	Retrospective, population-based cohort study	81,124	1960–1999	59,519 CIN 3 21,605 CC	NI	23 AC after CIN 3 18 AC after CC	AC after CIN 3: SIR = 5.9 AC after CC: SIR = 6.3	L
7.	Gaudet et al. (2014) [22]	British Columbia (Canada)	Retrospective, population-based cohort study	54,320	1985–2005	54,320 CIN 2 and CIN 3 ***	35	4 AC after CIN 2 16 AC after CIN 3	AC after CIN 2: SIR = 0.89 (95% Cl: 0.09–3.35) AC after CIN 3: SIR = 2.28 (95% Cl: 0.71–5.42)	L
8.	Hemminki et al. (2001) [23]	Sweden	Retrospective, population-based cohort study	17,234	1958–1996	17,234 CC	NI	16 AC	AC after CC: SIR = 4.22 (95% Cl: 2.41–6.55)	L
9.	Hemminki et al. (2000) [24]	Sweden	Retrospective, population-based cohort study	135,386	1958–1996	117,830 CIN 3 17,556 CC	NI	68 AC after CIN 3 17 AC after CC	AC after CIN 3: SIR = 3.75 (95% Cl: 2.91–4.69) AC after CC: SIR = 3.92 (95% Cl: 2.28–6.00)	L
10.	Heráclio et al. (2018) [25]	Recife (Brazil)	Prospective, cross-sectional study	324	2008–2009	200 CIN 1, 97 CIN 2 or CIN 3, 27 CC	33	37 AIN	AIN after CIN 1: IR = 7% AIN after CIN 2/3: IR = 18.5%	L
11.	Jakobsson et al. (2011) [26]	Finland	Retrospective, population-based cohort study	26,876	1987–2006	26,876 CIN (unknown grade)	NI	3 AC	AC after CIN: SIR = 3.56 (95% Cl: 0.73–10.4)	H
12.	Jiménez et al. (2009) [27]	Ontario (Canada)	Retrospective, population-based cohort study	674	1992–2005	7 CCs, 3 VaC, and 1 VC	61	674 AC	AC after HPV-RGD: OR = 10.5 (95% CI: 3.6–30.3) AC after CC: OR = 6.84 (95% CI: 2.16–21.61)	H
13.	Kalliala et al. (2005) [28]	Helsinki (Finland)	Retrospective cohort study	7564	1974–2003	2446 CIN 1 1543 CIN 2 1334 CIN 3 2241 CIN “not otherwise specified”	NI	3 AC	AC after CIN: SIR = 5.7 (95% Cl: 1.2 to 17.0)	L
14.	Matsuo et al. (2018) [29]	USA *	Retrospective, population-based cohort study	79,050	1973–2013	79,050 CC	63	49 AC	10-, 20-, 30-year cumulative incidence for AC after CC: 0.04%, 0.16%, and 0.38%	H
15.	Neumann et al. (2016) [30]	France ****	Retrospective, population-based cohort study	4808	1989–2007	4234 CC 339 VC 235 VaC	NI	5 AC after CC 1 AC after VC 0 AC after VaC	AC after CC: SIR = 5.42 (95% Cl: 1.75–12.64) AC after VC: SIR = 11.7 (95% Cl: 0.15–65.51)	L
16.	Pan et al. (2019) [31]	Scotland	Retrospective, population-based cohort study	NI	1989–2015	69,714 CIN 3	30	37 AC after CIN 3	AC after CIN 3: SIR = 2.6 (95% Cl: 1.9–3.6)	L
17.	Papatla et al. (2019) [32]	USA *	Retrospective, population-based cohort study	21,060	1973–2014	21,060 CC	61.73	17 AC	AC after CC: SIR = 2.20 (95% Cl: 1.28–3.52)	L
18.	Preti et al. (2020) [33]	Piedmont (Italy)	Retrospective, population-based cohort study	3184	1992–2004	3184 CIN 2 or 3	NI	1 AC	AC after CIN 2 or 3: SIR = 1.8 (95% Cl: 0.04–10.0)	H
19.	Rabkin et al. (1992) [34]	USA *	Retrospective, population-based cohort study	25,295	1935–1988	25,295 CC	NI	12 AC	AC after CC: SIR = 4.6 (95% Cl: 2.4–8.1)	H
20.	Saleem et al. (2011) [35]	USA *	Retrospective, population-based cohort study	189,206	1973–2007	124,075 CIN 3 6792 VIN 3 1463 VaIN 3 43,669 CC 9950 VC 3257 VaC	NI	255 AC	AC after CIN 3: SIR = 16.4 (95% CI: 13.7–19.2) AC after CC: SIR = 6.2 (95% CI: 4.1–8.7) AC after VIN 3: SIR = 22.2 (95% CI: 16.7–28.4) AC after VC: SIR = 17.4 (95% CI: 11.5–24.4) AC after VaIN 3: SIR = 7.6 (95% CI: 2.4–15.6) AC after VaC: SIR = 1.8 (95% CI: 0.2–5.3)	L
21.	Sand et al. (2016) [36]	Denmark	Retrospective, population-based cohort study	156,290	1978–2012	52,135 CIN 2 104,155 CIN 3	33.8 for CIN 2 34.0 for CIN 3	32 AC after CIN 2 125 AC after CIN 3	AC after CIN 2: SIR = 2.9 (2.0–4.1) AC after CIN 3: SIR = 4.2 (3.4–5.0)	L
22.	Suk et al. (2018) [37]	USA *	Retrospective, population-based cohort study	52,589	1973–2014	44,011 CC 6905 VC 1673 VaC	63 for CC, 61 for VC, 95 for VaC	34 AC after CC 31 AC after VC 1 AC after VaC	AC after CC: SIR = 2.3 (95% CI: 1.6–3.2) AC after VC: SIR = 13.2 (95% CI: 8.9–18.7) AC after VaC: SIR = 2.3 (95% CI: 0.1–12.8)	L
23.	Tatti et al. (2012) [38]	Buenos Aires (Argentina)	Prospective, cross-sectional study	481	2005–2011	121 CIN 1 114 CIN 2/3 188 VIN 1 39 VIN 2/3 70 VaIN 1 22 VaIN 2/3	35	28 AIN 2/3 106 AIN 1	No information (AIN after CIN 2/3 comparted to AIN after CIN 1: OR = 1.91)	H
24.	Tomassi et al. (2018) [39]	Southern California (USA)	Retrospective, population-based cohort study	221,511	2005–2015	1168 CC 15,711 CIN 2/3 109,893 CIN 1 94,739 genital warts	63.8	1 AC after CC 5 AC after CIN 2/3 14 AC after CIN 1 14 AC after genital warts	AC after CC: IR = 0.09% AC after CIN 2/3: IR = 0.03% AC after CIN 1: IR = 0.01% AC after genital warts: IR = 0.01%	H
25.	Wang et al. (2020) [40]	USA *	Retrospective, population-based cohort study	56,127	2000–2015	46,550 CC 7855 VC 1722 VaC	NI	50 AC after CC 9 AC after VC 1 AC after VaC	AC after CC: SIR = 1.63 (95% Cl: 1.21–2.14) AC after VC: SIR = 1.10 (95% Cl: 0.50–2.09) AC after VaC: SIR = 0.62 (95% Cl: 0.01–3.47)	L

* Nine areas of the United States covered by the National Cancer Institute’s Surveillance, Epidemiology, and End Results (SEER) Program (Connecticut, Hawaii, Iowa, New Mexico, and Utah, and the metropolitan areas of San Francisco–Oakland, Detroit, Seattle–Puget Sound, and Atlanta. ** One out of five cancer registries divided results for AC and rectal cancer; therefore, only data from US SEER program are included. *** No information about separate numbers of CIN 2 and CIN 3. **** Registries cover eight administrative regions of France (Bas-Rhin, Calvados, Doubs, Hérault, Isère, Manche, Somme, and Tarn), which comprise six million inhabitants, representing 9.6% of the French metropolitan population. AC—anal cancer; AIN—anal intraepithelial neoplasia; CC—cervical cancer; VC—vulvar cancer; VaC—vaginal cancer; CIN—cervical intraepithelial neoplasia; VIN—vulvar intraepithelial neoplasia; VaIN—vaginal intraepithelial neoplasia; L—low risk of bias; H—high risk of bias; U—unclear risk of bias; NI—no information; SIR—standardized incidence ratio; IR—incidence risk; OR—odds ratio.

**Table 2:** Incidence rate and incidence rate per 100,000 PY of anal intraepithelial neoplasia or anal cancer presented in reviewed articles or calculated on the basis of published data.

No.	Authors and Year of Publication	Number of Secondary AIN or AC/Number of Primary HPV-RGD	Person–Years	Incidence Rate of AIN or AC	IR per 100,000 Person–Years	Comment
1.	Acevedo-Fontánez et al. (2018) [16]	10 AC/8039 CC 3 AC/1378 VC 1 AC/773 VaC	119,617 14,631 6554	0.124% 0.217% 0.129%	8.36 20.5 15.26	
2.	Chaturvedi et al. (2007) [17]	-	-	-	-	No information about the number of AC cases.
3.	Ebisch et al. (2017) [18]	73 AC/89,018 CIN 3 80 AIN 3/89,018 CIN 3	1,261,804 1,261,804	0.082% 0.090%	5.79 6.34	
4.	Edgren et al. (2007) [19]	131 AC/125,292 CIN 3	2,193,409	0.105%	5.97	
5.	ElNaggar et al. (2013) [20]	13 AIN/90 CIN 48 AIN/132 VIN 10 AIN/55 VaIN	- - -	14.4% 36.4% 18.2%	- - -	1 AIN out of 3 CC, but not included because of the small number of cases.
6.	Evans et al. (2003) [21]	23 AC/59,519 CIN 3 18 AC/21,605 CC	477,069 145,621	0.039% 0.083%	4.82 12.36	
7.	Gaudet et al. (2014) [22]	20 AC/54,320 CIN 2 and CIN 3	545,945	0.037%	3.66	
8.	Hemminki et al. (2001) [23]	16 AC/17,234 CC	-	0.093%	-	
9.	Hemminki et al. (2000) [24]	68 AC/117,830 CIN 3 17 AC/17,556 CC	-	0.058% 0.097%	-	
10.	Heráclio et al. (2018) [25]	14 AIN/200 CIN 1 23 AIN/124 CIN 2 and 3	-	7% 18.5%	-	
11.	Jakobsson et al. (2011) [26]	3 AC/26,876 CIN	226,510	0.011%	1.32	
12.	Jiménez et al. (2009) [27]	-	-	-	-	No information about the total number of CC, VC, or VaC cases.
13.	Kalliala et al. (2005) [28]	3 AC/7564 CIN	97,556	0.040%	3.08	
14.	Matsuo et al. (2018) [29]	49 AC/79,050 CC	-	0.062%	-	
15.	Neumann et al. (2016) [30]	3 AC/4234 CC 1 AC/339 VC	28,122 1533	0.071% 0.295%	10.67 65.23	
16.	Pan et al. (2019) [31]	37 AC/69,714 CIN 3	893,622	0.053%	4.14	
17.	Papatla et al. (2019) [32]	17 AC/21,060 CC	-	0.081%	-	
18.	Preti et al. (2020) [33]	1 AC/3184 CIN 2 and 3	20,022	0.031%	4.99	
19.	Rabkin et al. (1992) [34]	12 AC/25,295 CC	156,838	0.047%	7.65	
20.	Saleem et al. (2011) [35]	137 AC/124,075 CIN 3 28 AC/43,669 CC 5 AC/1463 VaIN 3 2 AC/3257 VaC 55 AC/6792 VIN 3 28 AC/9950 VC	-	0.110% 0.064% 0.342% 0.061% 0.810% 0.281%	-	
21.	Sand et al. (2016) [36]	32 AC/52,135 CIN 2 125 AC/104,155 CIN 3	597,467 1,529,564	0.061% 0.120%	5.36 8.17	
22.	Suk et al. (2018) [37]	34 AC/44,011 CC 31 AC/6905 VC 1 AC/1673 VaC	473,820 48,373 9057	0.077% 0.449% 0.060%	7.18 64.09 11.04	
23.	Tatti et al. (2012) [38]	20 AIN/114 CIN 2 and 3 35 AIN/121 CIN 1 18 AIN/39 VIN 7 AIN/22 VaIN 2 and 3 27 AIN/70 VaIN 1	-	17.544% 28.926% 46.154% 31.818% 38.571%	-	Results without dividing AIN into HSIL (AIN 2/3) and LSIL (AIN 1).
24.	Tomassi et al. (2018) [39]	1 AC/1168 CC 14 AC/109,893 CIN 1 5 AC/15,711 CIN 2 and 3	10,359 708,690 114,031	0.086% 0.013% 0.032%	9.65 1.98 4.38	
25.	Wang et al. (2020) [40]	50 AC/46,550 CC 9 AC/7855 VC 1 AC/1722 VaC	- - -	0.107% 0.115% 0.058%	7.6 2.1 8.3	No information about PY. IR per 100,000 PY as provided by the authors of the publication.

AC—anal cancer; AIN—anal intraepithelial neoplasia; CC—cervical cancer; VC—vulvar cancer; VaC—vaginal cancer; CIN—cervical intraepithelial neoplasia; VIN—vulvar intraepithelial neoplasia; VaIN—vaginal intraepithelial neoplasia.

**Table 3:** Standardized incidence ratio (SIR), incidence rate (IR) and incidence rate per 100,000 person–years (IR per 100,000 PY) of anal cancer (AC) and anal intraepithelial neoplasia (AIN) among patients diagnosed and treated for gynecological HPV-related diseases—a summary of literature reviews.

Type of HPV-Related Gynecological Disease	Risk of AC Mean SIR (95% Cl) ^1^	Risk of AC Mean IR (95% Cl) ^2^	Risk of AC Mean IR per 100,000 PY (95% Cl) ^3^	Risk of AIN Mean SIR (95% Cl) ^1^	Risk of AIN Mean IR (95% Cl) ^2^	Risk of AIN Mean IR per 100,000 PY (95% Cl) ^3^
**Cervical** **cancer**	3.814 (1.21–6.41)	0.086% (0.07–0.102)	9.73 (8.03–11.43)			
**Vulvar** **cancer**	14.55 (0.15–24.4)	0.265% (0.17–0.36)	37.98 (22.64–53.32)			
**Vaginal** **cancer**	1.8 (0.2–5.3)	0.096% (0.009–0.183)	11.78 (0–29.15)			
**CIN 3**	5.701 (2.23–19.2)	0.084% (0.076–0.092)	5.78 (4.85–5.89)	6.68 (3.64–12.25)		6.34 (5.10–7.90)
**CIN (1–3)**	4.563 (0.12–19.2)	0.066% (0.06–0.072)	5.37 (4.85–5.89)		16.45% (13.25–19.65)	
**VIN 3**		0.810% (0.59–1.03)				
**VIN (1–3)**					36.4% (28–44.8)	
**VaIN 3**		0.342% (0.037–0.647)				
**VaIN (1–3)**					18.2% (7.8–28.6)	

^1^ Note: SIR data based on all reviewed literature. If SIR = 1, it means that there is no difference between the population of interest (here, women with specific HPV-related gynecological disease) and the general population. If SIR > 1, it means that there is higher risk of the disease in the population of interest than in the general population. ^2^ Note: IR data based on all reviewed literature. If IR = 0.084%, it means that out of 100,000 people with specific HPV-related gynecological disease, 84 will develop anal cancer/anal intraepithelial neoplasm. ^3^ Note: IR per 100,000 PY data based on all reviewed literature. It is a rate of newly diagnosed cases of AC/AIN in a cohort per 100,000 person–years of observation time.

## Data Availability

Not applicable.

## Supplementary Materials

The following supporting information can be downloaded at: https://www.mdpi.com/article/10.3390/jcm12134216/s1. References [80,82] are cited in the supplementary materials.

## Abbreviations

AC	Anal cancer
AIN	Anal intraepithelial neoplasia
CC	Cervical cancer
CIN	Cervical intraepithelial neoplasia
Cl	Confidence level
HPV	Human papillomavirus
HPV-RGD	HPV-related gynecological diseases
IR	Incidence risk
OR	Odds ratio
PY	Person–years
SD	Standard deviation
SIR	Standardized incidence ratio
VaC	Vaginal cancer
VaIN	Vaginal intraepithelial neoplasia
VIN	Vulvar intraepithelial neoplasia
VC	Vulvar cancer

## References

[1] Sung H., Ferlay J., Siegel R.L., Laversanne M., Soerjomataram I., Jemal A., Bray F. (2021). Global Cancer Statistics 2020: GLOBOCAN Estimates of Incidence and Mortality Worldwide for 36 Cancers in 185 Countries. CA. Cancer J. Clin..

[2] D’Souza G., Wiley D.J., Li X., Chmiel J.S., Margolick J.B., Cranston R.D., Jacobson L.P. (2008). Incidence and Epidemiology of Anal Cancer in the Multicenter AIDS Cohort Study. J. Acquir. Immune Defic. Syndr. (1999).

[3] Silverberg M.J., Lau B., Justice A.C., Engels E., Gill M.J., Goedert J.J., Kirk G.D., D’Souza G., Bosch R.J., Brooks J.T. (2012). Risk of Anal Cancer in HIV-Infected and HIV-Uninfected Individuals in North America. Clin. Infect. Dis. Off. Publ. Infect. Dis. Soc. Am..

[4] Shiels M.S., Pfeiffer R.M., Chaturvedi A.K., Kreimer A.R., Engels E.A. (2012). Impact of the HIV Epidemic on the Incidence Rates of Anal Cancer in the United States. J. Natl. Cancer Inst..

[5] de Sanjosé S., Bruni L., Alemany L. (2014). HPV in Genital Cancers (at the Exception of Cervical Cancer) and Anal Cancers. Presse Med..

[6] de Martel C., Plummer M., Vignat J., Franceschi S. (2017). Worldwide Burden of Cancer Attributable to HPV by Site, Country and HPV Type. Int. J. Cancer.

[7] Gheit T. (2019). Mucosal and Cutaneous Human Papillomavirus Infections and Cancer Biology. Front. Oncol..

[8] Ibeanu O.A. (2011). Molecular Pathogenesis of Cervical Cancer. Cancer Biol. Ther..

[9] Hernandez B.Y., McDuffie K., Zhu X., Wilkens L.R., Killeen J., Kessel B., Wakabayashi M.T., Bertram C.C., Easa D., Ning L. (2005). Anal Human Papillomavirus Infection in Women and Its Relationship with Cervical Infection. Cancer Epidemiol. Biomark. Prev..

[10] Jacot-Guillarmod M., Balaya V., Mathis J., Hübner M., Grass F., Cavassini M., Sempoux C., Mathevet P., Pache B. (2022). Women with Cervical High-Risk Human Papillomavirus: Be Aware of Your Anus! The ANGY Cross-Sectional Clinical Study. Cancers.

[11] Clarke M.A., Wentzensen N. (2018). Strategies for Screening and Early Detection of Anal Cancers: A Narrative and Systematic Review and Meta-Analysis of Cytology, HPV Testing, and Other Biomarkers. Cancer Cytopathol..

[12] Lin C., Slama J., Gonzalez P., Goodman M.T., Xia N., Kreimer A.R., Wu T., Hessol N.A., Shvetsov Y., Ortiz A.P. (2019). Cervical Determinants of Anal HPV Infection and High-Grade Anal Lesions in Women: A Collaborative Pooled Analysis. Lancet Infect. Dis..

[13] Page M.J., McKenzie J.E., Bossuyt P.M., Boutron I., Hoffmann T.C., Mulrow C.D., Shamseer L., Tetzlaff J.M., Akl E.A., Brennan S.E. (2021). The PRISMA 2020 Statement: An Updated Guideline for Reporting Systematic Reviews. BMJ.

[14] Waxman A.G., Chelmow D., Darragh T.M., Lawson H., Moscicki A.-B. (2012). Revised Terminology for Cervical Histopathology and Its Implications for Management of High-Grade Squamous Intraepithelial Lesions of the Cervix. Obstet. Gynecol..

[15] Whiting P.F., Rutjes A.W.S., Westwood M.E., Mallett S., Deeks J.J., Reitsma J.B., Leeflang M.M.G., Sterne J.A.C., Bossuyt P.M.M. (2011). QUADAS-2 Group QUADAS-2: A Revised Tool for the Quality Assessment of Diagnostic Accuracy Studies. Ann. Intern. Med..

[16] Acevedo-Fontánez A.I., Suárez E., Torres Cintrón C.R., Ortiz A.P. (2018). Risk of Anal Cancer in Women with a Human Papillomavirus–Related Gynecological Neoplasm: Puerto Rico 1987–2013. J. Low. Genit. Tract Dis..

[17] Chaturvedi A.K., Engels E.A., Gilbert E.S., Chen B.E., Storm H., Lynch C.F., Hall P., Langmark F., Pukkala E., Kaijser M. (2007). Second Cancers Among 104760 Survivors of Cervical Cancer: Evaluation of Long-Term Risk. JNCI J. Natl. Cancer Inst..

[18] Ebisch R.M.F., Rutten D.W.E., IntHout J., Melchers W.J.G., Massuger L.F.A.G., Bulten J., Bekkers R.L.M., Siebers A.G. (2017). Long-Lasting Increased Risk of Human Papillomavirus–Related Carcinomas and Premalignancies After Cervical Intraepithelial Neoplasia Grade 3: A Population-Based Cohort Study. J. Clin. Oncol..

[19] Edgren G., Sparén P. (2007). Risk of Anogenital Cancer after Diagnosis of Cervical Intraepithelial Neoplasia: A Prospective Population-Based Study. Lancet Oncol..

[20] ElNaggar A.C., Santoso J.T. (2013). Risk Factors for Anal Intraepithelial Neoplasia in Women with Genital Dysplasia. Obstet. Gynecol..

[21] Evans H.S., Newnham A., Hodgson S.V., Møller H. (2003). Second Primary Cancers after Cervical Intraepithelial Neoplasia III and Invasive Cervical Cancer in Southeast England. Gynecol. Oncol..

[22] Gaudet M., Hamm J., Aquino-Parsons C. (2014). Incidence of Ano-Genital and Head and Neck Malignancies in Women with a Previous Diagnosis of Cervical Intraepithelial Neoplasia. Gynecol. Oncol..

[23] Hemminki K., Jiang Y., Dong C. (2001). Second Primary Cancers after Anogenital, Skin, Oral, Esophageal and Rectal Cancers: Etiological Links?. Int. J. Cancer.

[24] Hemminki K., Dong C., Vaittinen P. (2000). Second Primary Cancer after in Situ and Invasive Cervical Cancer. Epidemiol. Camb. Mass.

[25] Heráclio S.A., De Souza A.S.R., De Souza P.R.E., Katz L., Lima Junior S.F., Amorim M.M.R. (2018). Cross-Sectional Study of Anal Intraepithelial Lesions in Women with Cervical Neoplasia without HIV. Int. J. Gynecol. Obstet..

[26] Jakobsson M., Pukkala E., Paavonen J., Tapper A., Gissler M. (2011). Cancer Incidence among Finnish Women with Surgical Treatment for Cervical Intraepithelial Neoplasia, 1987–2006. Int. J. Cancer.

[27] Jiménez W., Paszat L., Kupets R., Wilton A., Tinmouth J. (2009). Presumed Previous Human Papillomavirus (HPV) Related Gynecological Cancer in Women Diagnosed with Anal Cancer in the Province of Ontario. Gynecol. Oncol..

[28] Kalliala I., Anttila A., Pukkala E., Nieminen P. (2005). Risk of Cervical and Other Cancers after Treatment of Cervical Intraepithelial Neoplasia: Retrospective Cohort Study. BMJ.

[29] Matsuo K., Blake E.A., Machida H., Mandelbaum R.S., Roman L.D., Wright J.D. (2018). Incidences and Risk Factors of Metachronous Vulvar, Vaginal, and Anal Cancers after Cervical Cancer Diagnosis. Gynecol. Oncol..

[30] Neumann F., Jégu J., Mougin C., Prétet J.-L., Guizard A.-V., Lapôtre-Ledoux B., Bara S., Bouvier V., Colonna M., Troussard X. (2016). Risk of Second Primary Cancer after a First Potentially-Human Papillomavirus-Related Cancer: A Population-Based Study. Prev. Med..

[31] Pan J., Kavanagh K., Cuschieri K., Pollock K.G., Gilbert D.C., Millan D., Bell S., Graham S.V., Williams A.R.W., Cruickshank M.E. (2019). Increased Risk of HPV-Associated Genital Cancers in Men and Women as a Consequence of Pre-Invasive Disease. Int. J. Cancer.

[32] Papatla K., Halpern M.T., Hernandez E., Brown J., Benrubi D., Houck K., Chu C., Rubin S. (2019). Second Primary Anal and Oropharyngeal Cancers in Cervical Cancer Survivors. Am. J. Obstet. Gynecol..

[33] Preti M., Rosso S., Micheletti L., Libero C., Sobrato I., Giordano L., Busso P., Gallio N., Cosma S., Bevilacqua F. (2020). Risk of HPV-Related Extra-Cervical Cancers in Women Treated for Cervical Intraepithelial Neoplasia. BMC Cancer.

[34] Rabkin C.S., Biggar R.J., Melbye M., Curtis R.E. (1992). Second Primary Cancers Following Anal and Cervical Carcinoma: Evidence of Shared Etiologic Factors. Am. J. Epidemiol..

[35] Saleem A.M., Paulus J.K., Shapter A.P., Baxter N.N., Roberts P.L., Ricciardi R. (2011). Risk of Anal Cancer in a Cohort with Human Papillomavirus–Related Gynecologic Neoplasm. Obstet. Gynecol..

[36] Sand F.L., Munk C., Jensen S.M., Svahn M.F., Frederiksen K., Kjær S.K. (2016). Long-Term Risk for Noncervical Anogenital Cancer in Women with Previously Diagnosed High-Grade Cervical Intraepithelial Neoplasia: A Danish Nationwide Cohort Study. Cancer Epidemiol. Biomark. Prev..

[37] Suk R., Mahale P., Sonawane K., Sikora A.G., Chhatwal J., Schmeler K.M., Sigel K., Cantor S.B., Chiao E.Y., Deshmukh A.A. (2018). Trends in Risks for Second Primary Cancers Associated with Index Human Papillomavirus–Associated Cancers. JAMA Netw. Open.

[38] Tatti S., Suzuki V., Fleider L., Maldonado V., Caruso R. (2012). Anal Intraepithelial Lesions in Women with Human PapillomavirusYRelated Disease. J. Low Genit. Tract Dis..

[39] Tomassi M.J., Abbas M.A., Klaristenfeld D.D. (2019). Expectant Management Surveillance for Patients at Risk for Invasive Squamous Cell Carcinoma of the Anus: A Large US Healthcare System Experience. Int. J. Color. Dis..

[40] Wang M., Sharma A., Osazuwa-Peters N., Simpson M.C., Schootman M., Piccirillo J.F., Huh W.K., Adjei Boakye E. (2020). Risk of Subsequent Malignant Neoplasms after an Index Potentially-Human Papillomavirus (HPV)-Associated Cancers. Cancer Epidemiol..

[41] Saraiya M., Unger E.R., Thompson T.D., Lynch C.F., Hernandez B.Y., Lyu C.W., Steinau M., Watson M., Wilkinson E.J., Hopenhayn C. (2015). US Assessment of HPV Types in Cancers: Implications for Current and 9-Valent HPV Vaccines. JNCI J. Natl. Cancer Inst..

[42] Watson A.J.M., Smith B.B., Whitehead M.R., Sykes P.H., Frizelle F.A. (2006). Malignant Progression of Anal Intra-Epithelial Neoplasia. ANZ J. Surg..

[43] Kim Y.-T., Lee J.M., Hur S.-Y., Cho C.-H., Kim Y.T., Kim S.C., Kang S.B. (2010). Clearance of Human Papillomavirus Infection after Successful Conization in Patients with Cervical Intraepithelial Neoplasia. Int. J. Cancer.

[44] Santoso J.T., Long M., Crigger M., Wan J.Y., Haefner H.K. (2010). Anal Intraepithelial Neoplasia in Women with Genital Intraepithelial Neoplasia. Obstet. Gynecol..

[45] Clarke M.A., Deshmukh A.A., Suk R., Roberts J., Gilson R., Jay N., Stier E.A., Wentzensen N. (2022). A Systematic Review and Meta-analysis of Cytology and HPV-related Biomarkers for Anal Cancer Screening among Different Risk Groups. Int. J. Cancer.

[46] IANS—IANS Committees. https://iansoc.org/IANS-Committees.

[47] Muñoz N., Manalastas R., Pitisuttithum P., Tresukosol D., Monsonego J., Ault K., Clavel C., Luna J., Myers E., Hood S. (2009). Safety, Immunogenicity, and Efficacy of Quadrivalent Human Papillomavirus (Types 6, 11, 16, 18) Recombinant Vaccine in Women Aged 24–45 Years: A Randomised, Double-Blind Trial. Lancet Lond. Engl..

[48] Wei L., Xie X., Liu J., Zhao Y., Chen W., Zhao C., Wang S., Liao X., Shou Q., Qiu Y. (2019). Efficacy of Quadrivalent Human Papillomavirus Vaccine against Persistent Infection and Genital Disease in Chinese Women: A Randomized, Placebo-Controlled Trial with 78-Month Follow-Up. Vaccine.

[49] Bartels H.C., Postle J., Rogers A.C., Brennan D. (2020). Prophylactic Human Papillomavirus Vaccination to Prevent Recurrence of Cervical Intraepithelial Neoplasia: A Meta-Analysis. Int. J. Gynecol. Cancer Off. J. Int. Gynecol. Cancer Soc..

[50] Jentschke M., Kampers J., Becker J., Sibbertsen P., Hillemanns P. (2020). Prophylactic HPV Vaccination after Conization: A Systematic Review and Meta-Analysis. Vaccine.

[51] Kechagias K.S., Kalliala I., Bowden S.J., Athanasiou A., Paraskevaidi M., Paraskevaidis E., Dillner J., Nieminen P., Strander B., Sasieni P. (2022). Role of Human Papillomavirus (HPV) Vaccination on HPV Infection and Recurrence of HPV Related Disease after Local Surgical Treatment: Systematic Review and Meta-Analysis. BMJ.

[52] Goodman E., Reuschenbach M., Kaminski A., Ronnebaum S. (2022). Human Papillomavirus Vaccine Impact and Effectiveness in Six High-Risk Populations: A Systematic Literature Review. Vaccines.

[53] Drolet M., Bénard É., Pérez N., Brisson M. (2019). HPV Vaccination Impact Study Group Population-Level Impact and Herd Effects Following the Introduction of Human Papillomavirus Vaccination Programmes: Updated Systematic Review and Meta-Analysis. Lancet Lond. Engl..

[54] Kjaer S.K., Nygård M., Sundström K., Dillner J., Tryggvadottir L., Munk C., Berger S., Enerly E., Hortlund M., Ágústsson Á.I. (2020). Final Analysis of a 14-Year Long-Term Follow-up Study of the Effectiveness and Immunogenicity of the Quadrivalent Human Papillomavirus Vaccine in Women from Four Nordic Countries. EClinicalMedicine.

[55] Olsson S.-E., Restrepo J.A., Reina J.C., Pitisuttithum P., Ulied A., Varman M., Van Damme P., Moreira E.D., Ferris D., Block S. (2020). Long-Term Immunogenicity, Effectiveness, and Safety of Nine-Valent Human Papillomavirus Vaccine in Girls and Boys 9 to 15 Years of Age: Interim Analysis after 8 Years of Follow-Up. Papillomavirus Res. Amst. Neth..

[56] Lei J., Ploner A., Elfström K.M., Wang J., Roth A., Fang F., Sundström K., Dillner J., Sparén P. (2020). HPV Vaccination and the Risk of Invasive Cervical Cancer. N. Engl. J. Med..

[57] Falcaro M., Castañon A., Ndlela B., Checchi M., Soldan K., Lopez-Bernal J., Elliss-Brookes L., Sasieni P. (2021). The Effects of the National HPV Vaccination Programme in England, UK, on Cervical Cancer and Grade 3 Cervical Intraepithelial Neoplasia Incidence: A Register-Based Observational Study. Lancet Lond. Engl..

[58] Kjaer S.K., Dehlendorff C., Belmonte F., Baandrup L. (2021). Real-World Effectiveness of Human Papillomavirus Vaccination Against Cervical Cancer. J. Natl. Cancer Inst..

[59] Dehlendorff C., Baandrup L., Kjaer S.K. (2021). Real-World Effectiveness of Human Papillomavirus Vaccination Against Vulvovaginal High-Grade Precancerous Lesions and Cancers. J. Natl. Cancer Inst..

[60] Zhang L., Hemminki O., Chen T., Zheng G., Försti A., Sundquist K., Sundquist J., Hemminki K. (2019). Familial Clustering, Second Primary Cancers and Causes of Death in Penile, Vulvar and Vaginal Cancers. Sci. Rep..

[61] Herrero R., Quint W., Hildesheim A., Gonzalez P., Struijk L., Katki H.A., Porras C., Schiffman M., Rodriguez A.C., Solomon D. (2013). Reduced Prevalence of Oral Human Papillomavirus (HPV) 4 Years after Bivalent HPV Vaccination in a Randomized Clinical Trial in Costa Rica. PLoS ONE.

[62] Lehtinen M., Apter D., Eriksson T., Harjula K., Hokkanen M., Lehtinen T., Natunen K., Damaso S., Soila M., Bi D. (2020). Effectiveness of the AS04-Adjuvanted HPV-16/18 Vaccine in Reducing Oropharyngeal HPV Infections in Young Females-Results from a Community-Randomized Trial. Int. J. Cancer.

[63] Tsentemeidou A., Fyrmpas G., Stavrakas M., Vlachtsis K., Sotiriou E., Poutoglidis A., Tsetsos N. (2021). Human Papillomavirus Vaccine to End Oropharyngeal Cancer. A Systematic Review and Meta-Analysis. Sex. Transm. Dis..

[64] Hirsch B.E., McGowan J.P., Fine S.M., Vail R., Merrick S.T., Radix A., Hoffmann C.J., Gonzalez C.J. (2022). Screening for Anal Dysplasia and Cancer in Adults with HIV.

[65] Leeds I.L., Fang S.H. (2016). Anal Cancer and Intraepithelial Neoplasia Screening: A Review. World J. Gastrointest. Surg..

[66] Kelly H., Chikandiwa A., Alemany Vilches L., Palefsky J.M., de Sanjose S., Mayaud P. (2020). Association of Antiretroviral Therapy with Anal High-Risk Human Papillomavirus, Anal Intraepithelial Neoplasia, and Anal Cancer in People Living with HIV: A Systematic Review and Meta-Analysis. Lancet HIV.

[67] Albuquerque A., Stirrup O., Nathan M., Clifford G.M. (2020). Burden of Anal Squamous Cell Carcinoma, Squamous Intraepithelial Lesions and HPV16 Infection in Solid Organ Transplant Recipients: A Systematic Review and Meta-Analysis. Am. J. Transplant. Off. J. Am. Soc. Transplant. Am. Soc. Transpl. Surg..

[68] Garland S.M., Brotherton J.M.L., Moscicki A.B., Kaufmann A.M., Stanley M., Bhatla N., Sankaranarayanan R., de Sanjosé S., Palefsky J.M. (2017). IPVS HPV Vaccination of Immunocompromised Hosts. Papillomavirus Res. Amst. Neth..

[69] Perkins R.B., Guido R.S., Castle P.E., Chelmow D., Einstein M.H., Garcia F., Huh W.K., Kim J.J., Moscicki A.-B., Nayar R. (2020). 2019 ASCCP Risk-Based Management Consensus Guidelines for Abnormal Cervical Cancer Screening Tests and Cancer Precursors. J. Low. Genit. Tract Dis..

[70] Mugo N.R., Eckert L., Magaret A.S., Cheng A., Mwaniki L., Ngure K., Celum C., Baeten J.M., Galloway D.A., Wamalwa D. (2018). Quadrivalent HPV Vaccine in HIV-1-Infected Early Adolescent Girls and Boys in Kenya: Month 7 and 12 Post Vaccine Immunogenicity and Correlation with Immune Status. Vaccine.

[71] Mugo N., Eckert L.O., Odero L., Gakuo S., Ngure K., Celum C., Baeten J.M., Barnabas R.V., Wald A. (2021). Antibody Responses to Prophylactic Quadrivalent Human Papillomavirus Vaccine at 48 Months among HIV-Infected Girls and Boys Ages 9–14 in Kenya, Africa. Vaccine.

[72] Staadegaard L., Rönn M.M., Soni N., Bellerose M.E., Bloem P., Brisson M., Maheu-Giroux M., Barnabas R.V., Drolet M., Mayaud P. (2022). Immunogenicity, Safety, and Efficacy of the HPV Vaccines among People Living with HIV: A Systematic Review and Meta-Analysis. EClinicalMedicine.

[73] Winer R.L., Kiviat N.B., Hughes J.P., Adam D.E., Lee S.-K., Kuypers J.M., Koutsky L.A. (2005). Development and Duration of Human Papillomavirus Lesions, after Initial Infection. J. Infect. Dis..

[74] Zielinski G.D., Snijders P.J., Rozendaal L., Voorhorst F.J., van der Linden H.C., Runsink A.P., de Schipper F.A., Meijer C.J. (2001). HPV Presence Precedes Abnormal Cytology in Women Developing Cervical Cancer and Signals False Negative Smears. Br. J. Cancer.

[75] Deshmukh A.A., Suk R., Shiels M.S., Sonawane K., Nyitray A.G., Liu Y., Gaisa M.M., Palefsky J.M., Sigel K. (2020). Recent Trends in Squamous Cell Carcinoma of the Anus Incidence and Mortality in the United States, 2001–2015. J. Natl. Cancer Inst..

[76] Grulich A.E., Poynten I.M., Machalek D.A., Jin F., Templeton D.J., Hillman R.J. (2012). The Epidemiology of Anal Cancer. Sex. Health.

[77] Stier E.A., Goldstone S.E., Einstein M.H., Jay N., Berry J.M., Wilkin T., Lee J.Y., Darragh T.M., Da Costa M., Panther L. (2013). Safety and Efficacy of Topical Cidofovir to Treat High-Grade Perianal and Vulvar Intraepithelial Neoplasia in HIV-Positive Men and Women. AIDS Lond. Engl..

[78] Tranoulis A., Laios A., Mitsopoulos V., Lutchman-Singh K., Thomakos N. (2017). Efficacy of 5% Imiquimod for the Treatment of Vaginal Intraepithelial Neoplasia-A Systematic Review of the Literature and a Meta-Analysis. Eur. J. Obstet. Gynecol. Reprod. Biol..

[79] Palefsky J.M., Lee J.Y., Jay N., Goldstone S.E., Darragh T.M., Dunlevy H.A., Rosa-Cunha I., Arons A., Pugliese J.C., Vena D. (2022). Treatment of Anal High-Grade Squamous Intraepithelial Lesions to Prevent Anal Cancer. N. Engl. J. Med..

[80] Standardized Incidence Ratio (SIR). https://www.cdc.gov/nceh/cancer-environment/pdfs/standardized-incidence-ratio-fact-sheet-508.pdf.

[81] Cervical Cancer—Cancer Stat Facts. https://seer.cancer.gov/statfacts/html/cervix.html.

[82] https://www.cdc.gov/csels/dsepd/ss1978/lesson3/section2.html.

