# Supplementary material for: Anal Cancer and Anal Intraepithelial Neoplasia Risk among Patients Treated for HPV-Related Gynecological Diseases—A Systematic Review
Source: J Clin Med. 2023 Jun 22;12(13):4216. doi: 10.3390/jcm12134216 (PMC10342585; doi:10.3390/jcm12134216)
Supplement: Supplementary file 1 [file jcm-12-04216-s001.zip › jcm-2420440-supplementary.pdf]

Supplementary Material S1:

Specific words and phrases used during search of PubMed and EBSCO Discovery Service databases:

“papillomavirus” or „HPV” or „cervical cancer” or „cervical intraepithelial neoplasia” or „CIN” or „cervical dysplasia” or „HSIL” or „vulvar cancer” or „vulvar intraepithelial neoplasia” or „vulvar dysplasia” or „VIN” or „vaginal cancer” or „vaginal intraepithelial neoplasia” or „VAIN” or „vaginal dysplasia”

AND

„AIN” or „Anal intraepithelial neoplasia” or „anal dysplasia” or „anal precancer” or „anal cancer” or „anus neoplasms”

Supplementary Material S2:

No.	Authors and year of publication	Risk of bias				Applicability concerns			Cumulative risk of bias
		Patient selection	Index Test	Reference standard	Flow and Timing	Patient selection	Index Test	Reference standard	
1.	Acevedo-Fontánez et al. (2018) [1]	L	L	L	L	L	L	L	L
2.	Chaturvedi et al. (2007) [2]	L	L	L	L	L	L	L	L
3.	Ebisch et al. (2017) [3]	L	L	L	L	L	L	L	L
4.	Edgren et al. (2007) [4]	L	L	L	L	L	L	L	L
5.	ElNaggar et al. (2013) [5]	H	H	L	L	H	L	L	H

6.	Evans et al. (2003) [6]	L	L	L	L	L	L	L	L
7.	Gaudet et al. (2014) [7]	L	L	L	L	L	L	L	L
8.	Hemminki et al. (2001) [8]	L	L	L	L	L	L	L	L
9.	Hemminki et al. (2000) [9]	L	L	L	L	L	L	L	L
10.	Heráclio et al. (2018) [10]	L	L	L	L	L	L	L	L
11.	Jakobsson et al. (2011) [11]	L	L	L	L	H	L	L	H
12.	Jiménez et al. (2009) [12]	L	L	L	L	L	H	L	H
13.	Kalliala et al. (2005) [13]	L	L	L	L	L	L	L	L
14.	Matsuo et al. (2018) [14]	L	L	L	L	L	H	L	H
15.	Neumann et al. (2016) [15]	L	L	L	L	L	L	L	L
16.	Pan et al. (2019) [16]	L	L	L	L	L	L	L	L
17.	Papatla et al. (2019) [17]	L	L	L	L	L	L	L	L
18.	Preti et al. (2020) [18]	H	L	L	L	H	L	L	H
19.	Rabkin et al. (1992) [19]	L	L	L	H	L	L	L	H
20.	Saleem et al. (2011)[20]	L	L	L	L	L	L	L	L
21.	Sand et al. (2016) [21]	L	L	L	L	L	L	L	L
22.	Suk et al. (2018) [22]	L	L	L	L	L	L	L	L
23.	Tatti et al. (2012) [23]	H	L	L	L	L	H	L	H
24.	Tomassi et al. (2018) [24]	L	H	L	L	L	H	L	H
25.	Wang et al. (2020) [25]	L	L	L	L	L	L	L	L

Table S1: Risk of bias and applicability concerns of selected articles based on QUADAS-2 recommendations [26].

Supplementary Material S3:

Definition of standardized incidence ratio (SIR) by Centres for Disease Control and Prevention CDC [27]:

The SIR is an estimate of the number of cancer cases in a given population compared to what might be "expected" based on a comparison with the cancer experience in a larger population. Thus, the SIR is a ratio of the number of cancers observed compared to the number expected. If the observed number of cases equals the expected number of cases, the value of an SIR is 1.0. Sometimes we multiply the SIR by 100 for ease of reporting.

If SIR = 1 it means that there is no difference between population of interest and general population. If SIR > 1, it means that there is higher risk of the disease in the population of interest than in the general population. If SIR of disease "A" is 6.5 and SIR of disease "B" is 2.4 it means that the risk of developing disease "A" is higher than the risk of developing disease "B".

Standardizing is a way to adjust a rate by taking into account factors about a population. For example, rates can be adjusted for factors such as age, sex, race, or ethnicity. In cancer analyses, adjusting for age is important because age is a risk factor for many cancers. Therefore, if the comparison population is composed of an older population, you might expect cancer to occur more frequently because cancer rates increase with age. In this example, the calculation of the SIR would take the age of the population into account and adjusts the comparison based upon the age of that population. We call this standardizing by age.

Supplementary Material S4:

Definition of incidence risk (IR) by Centres for Disease Control and Prevention CDC [28]:

Incidence risk is the proportion of an initially disease-free population that develops disease, becomes injured, or dies during a specified (usually limited) period of time. Synonyms include attack rate, incidence proportion, probability of getting disease, and cumulative incidence. Incidence risk is a proportion because the persons in the numerator, those who develop disease, are all included in the denominator (the entire population). Method for calculating:

IR= [number of new cases of disease during specified period] / [size of population at start of period]

If IR= 0.1% it means that out of 100,000 people of specific subpopulation 100 will develop a defined disease. Thus, if IR of disease "A" is 1% and IR of disease "B" is 0.1% it means that the risk of developing disease "A" is 10 times higher than the risk of developing disease "B".

Supplementary Material S5:

Definition of incidence risk per 100,000 person years (IR per 100,000 PY) by Centres for Disease Control and Prevention CDC [28]:

IR per 1000,000 PY is generally calculated from a long-term cohort follow-up study, wherein enrollees are followed over time and the occurrence of new cases of disease is documented. Typically, each person is observed from an established starting time until one of four "end points" is reached: onset of disease, death, migration out of the study ("lost to follow-up"), or the end of the study. Similar to the incidence proportion, the numerator of the incidence rate is the number of new cases identified during the period of observation. However, the denominator differs. The denominator is the sum of the time each person was observed, totaled for all persons. This denominator represents the total time the population was at risk of and being watched for disease. Thus, the incidence rate is the ratio of the number of cases to the total time the population is at risk of disease. Method for calculating:

IR per 1000,000 PY = [number of new cases of disease or injury during specified period] / [time each person was observed, totaled for all persons- in this case for 100,000 PY]

References

1. Acevedo-Fontánez, A.I.; Suárez, E.; Torres Cintrón, C.R.; Ortiz, A.P. Risk of Anal Cancer in Women With a Human Papillomavirus–Related Gynecological Neoplasm: Puerto Rico 1987–2013. *J. Low. Genit. Tract Dis.* **2018**, *22*, 225–230, doi:10.1097/LGT.0000000000000395.
2. Chaturvedi, A.K.; Engels, E.A.; Gilbert, E.S.; Chen, B.E.; Storm, H.; Lynch, C.F.; Hall, P.; Langmark, F.; Pukkala, E.; Kaijser, M.; et al. Second Cancers Among 104760 Survivors of Cervical Cancer: Evaluation of Long-Term Risk. *JNCI J. Natl. Cancer Inst.* **2007**, *99*, 1634–1643, doi:10.1093/jnci/djm201.

3. Ebisch, R.M.F.; Rutten, D.W.E.; IntHout, J.; Melchers, W.J.G.; Massuger, L.F.A.G.; Bulten, J.; Bekkers, R.L.M.; Siebers, A.G. Long-Lasting Increased Risk of Human Papillomavirus–Related Carcinomas and Premalignancies After Cervical Intraepithelial Neoplasia Grade 3: A Population-Based Cohort Study. *J. Clin. Oncol.* **2017**, *35*, 2542–2550, doi:10.1200/JCO.2016.71.4543.
4. Edgren, G.; Sparén, P. Risk of Anogenital Cancer after Diagnosis of Cervical Intraepithelial Neoplasia: A Prospective Population-Based Study. *Lancet Oncol.* **2007**, *8*, 311–316, doi:10.1016/S1470-2045(07)70043-8.
5. ElNaggar, A.C.; Santoso, J.T. Risk Factors for Anal Intraepithelial Neoplasia in Women With Genital Dysplasia. *Obstet. Gynecol.* **2013**, *122*, 218–223, doi:10.1097/AOG.0b013e31829a2ace.
6. Evans, H.S.; Newnham, A.; Hodgson, S.V.; Møller, H. Second Primary Cancers after Cervical Intraepithelial Neoplasia III and Invasive Cervical Cancer in Southeast England. *Gynecol. Oncol.* **2003**, *90*, 131–136, doi:10.1016/S0090-8258(03)00231-2.
7. Gaudet, M.; Hamm, J.; Aquino-Parsons, C. Incidence of Ano-Genital and Head and Neck Malignancies in Women with a Previous Diagnosis of Cervical Intraepithelial Neoplasia. *Gynecol. Oncol.* **2014**, *134*, 523–526, doi:10.1016/j.ygyno.2014.07.088.
8. Hemminki, K.; Jiang, Y.; Dong, C. Second Primary Cancers after Anogenital, Skin, Oral, Esophageal and Rectal Cancers: Etiological Links? *Int. J. Cancer* **2001**, *93*, 294–298, doi:10.1002/ijc.1319.
9. Hemminki, K.; Dong, C.; Vaittinen, P. Second Primary Cancer after in Situ and Invasive Cervical Cancer. *Epidemiol. Camb. Mass* **2000**, *11*, 457–461, doi:10.1097/00001648-200007000-00016.
10. Heráclio, S.A.; De Souza, A.S.R.; De Souza, P.R.E.; Katz, L.; Lima Junior, S.F.; Amorim, M.M.R. Cross-Sectional Study of Anal Intraepithelial Lesions in Women with Cervical Neoplasia without HIV. *Int. J. Gynecol. Obstet.* **2018**, *140*, 233–240, doi:10.1002/ijgo.12367.
11. Jakobsson, M.; Pukkala, E.; Paavonen, J.; Tapper, A.; Gissler, M. Cancer Incidence among Finnish Women with Surgical Treatment for Cervical Intraepithelial Neoplasia, 1987–2006. *Int. J. Cancer* **2011**, *128*, 1187–1191, doi:10.1002/ijc.25428.
12. Jiménez, W.; Paszat, L.; Kupets, R.; Wilton, A.; Tinmouth, J. Presumed Previous Human Papillomavirus (HPV) Related Gynecological Cancer in Women Diagnosed with Anal Cancer in the Province of Ontario. *Gynecol. Oncol.* **2009**, *114*, 395–398, doi:10.1016/j.ygyno.2009.05.006.
13. Kalliala, I.; Anttila, A.; Pukkala, E.; Nieminen, P. Risk of Cervical and Other Cancers after Treatment of Cervical Intraepithelial Neoplasia: Retrospective Cohort Study. *BMJ* **2005**, *331*, 1183–1185, doi:10.1136/bmj.38663.459039.7C.
14. Matsuo, K.; Blake, E.A.; Machida, H.; Mandelbaum, R.S.; Roman, L.D.; Wright, J.D. Incidences and Risk Factors of Metachronous Vulvar, Vaginal, and Anal Cancers after Cervical Cancer Diagnosis. *Gynecol. Oncol.* **2018**, *150*, 501–508, doi:10.1016/j.ygyno.2018.07.016.
15. Neumann, F.; Jégu, J.; Mougin, C.; Prétet, J.-L.; Guizard, A.-V.; Lapôtre-Ledoux, B.; Bara, S.; Bouvier, V.; Colonna, M.; Troussard, X.; et al. Risk of Second Primary Cancer after a First Potentially-Human Papillomavirus-Related Cancer: A Population-Based Study. *Prev. Med.* **2016**, *90*, 52–58, doi:10.1016/j.ypmed.2016.06.041.

16. Pan, J.; Kavanagh, K.; Cuschieri, K.; Pollock, K.G.; Gilbert, D.C.; Millan, D.; Bell, S.; Graham, S.V.; Williams, A.R.W.; Cruickshank, M.E.; et al. Increased Risk of HPV-Associated Genital Cancers in Men and Women as a Consequence of Pre-Invasive Disease. *Int. J. Cancer* **2019**, *145*, 427–434, doi:10.1002/ijc.32126.
17. Papatla, K.; Halpern, M.T.; Hernandez, E.; Brown, J.; Benrubi, D.; Houck, K.; Chu, C.; Rubin, S. Second Primary Anal and Oropharyngeal Cancers in Cervical Cancer Survivors. *Am. J. Obstet. Gynecol.* **2019**, *221*, 478.e1-478.e6, doi:10.1016/j.ajog.2019.05.025.
18. Preti, M.; Rosso, S.; Micheletti, L.; Libero, C.; Sobrato, I.; Giordano, L.; Busso, P.; Gallio, N.; Cosma, S.; Bevilacqua, F.; et al. Risk of HPV-Related Extra-Cervical Cancers in Women Treated for Cervical Intraepithelial Neoplasia. *BMC Cancer* **2020**, *20*, 972, doi:10.1186/s12885-020-07452-6.
19. Rabkin, C.S.; Biggar, R.J.; Melbye, M.; Curtis, R.E. Second Primary Cancers Following Anal and Cervical Carcinoma: Evidence of Shared Etiologic Factors. *Am. J. Epidemiol.* **1992**, *136*, 54–58, doi:10.1093/oxfordjournals.aje.a116420.
20. Saleem, A.M.; Paulus, J.K.; Shapter, A.P.; Baxter, N.N.; Roberts, P.L.; Ricciardi, R. Risk of Anal Cancer in a Cohort With Human Papillomavirus–Related Gynecologic Neoplasm. *Obstet. Gynecol.* **2011**, *117*, 643–649, doi:10.1097/AOG.0b013e31820bfb16.
21. Sand, F.L.; Munk, C.; Jensen, S.M.; Svahn, M.F.; Frederiksen, K.; Kjær, S.K. Long-Term Risk for Noncervical Anogenital Cancer in Women with Previously Diagnosed High-Grade Cervical Intraepithelial Neoplasia: A Danish Nationwide Cohort Study. *Cancer Epidemiol. Biomarkers Prev.* **2016**, *25*, 1090–1097, doi:10.1158/1055-9965.EPI-15-1291.
22. Suk, R.; Mahale, P.; Sonawane, K.; Sikora, A.G.; Chhatwal, J.; Schmeler, K.M.; Sigel, K.; Cantor, S.B.; Chiao, E.Y.; Deshmukh, A.A. Trends in Risks for Second Primary Cancers Associated With Index Human Papillomavirus–Associated Cancers. *JAMA Netw. Open* **2018**, *1*, e181999, doi:10.1001/jamanetworkopen.2018.1999.
23. Tatti, S.; Suzuki, V.; Fleider, L.; Maldonado, V.; Caruso, R. Anal Intraepithelial Lesions in Women With Human Papillomavirus Related Disease. *J Low Genit Tract Dis* **2012**; 16: 454-459 doi:10.1097/LGT.0b013e31825d2d7a.
24. Tomassi, M.J.; Abbas, M.A.; Klaristenfeld, D.D. Expectant Management Surveillance for Patients at Risk for Invasive Squamous Cell Carcinoma of the Anus: A Large US Healthcare System Experience. *Int. J. Colorectal Dis.* **2019**, *34*, 47–54, doi:10.1007/s00384-018-3167-7.
25. Wang, M.; Sharma, A.; Osazuwa-Peters, N.; Simpson, M.C.; Schootman, M.; Piccirillo, J.F.; Huh, W.K.; Adjei Boakye, E. Risk of Subsequent Malignant Neoplasms after an Index Potentially-Human Papillomavirus (HPV)-Associated Cancers. *Cancer Epidemiol.* **2020**, *64*, 101649, doi:10.1016/j.canep.2019.101649.
26. Whiting, P.F.; Rutjes, A.W.S.; Westwood, M.E.; Mallett, S.; Deeks, J.J.; Reitsma, J.B.; Leeflang, M.M.G.; Sterne, J.A.C.; Bossuyt, P.M.M.; QUADAS-2 Group QUADAS-2: A Revised Tool for the Quality Assessment of Diagnostic Accuracy Studies. *Ann. Intern. Med.* **2011**, *155*, 529–536, doi:10.7326/0003-4819-155-8-201110180-00009.
27. https://www.cdc.gov/nceh/cancer-environment/pdfs/standardized-incidence-ratio-fact-sheet-508.pdf (accessed on 10 May 2023).

28. https://www.cdc.gov/csels/dsepd/ss1978/lesson3/section2.html (accessed on 10 May 2023).
